# Draft Genome Sequences of *Carboxydothermus pertinax* and *C. islandicus*, Hydrogenogenic Carboxydotrophic Bacteria

**DOI:** 10.1128/genomeA.01648-16

**Published:** 2017-02-23

**Authors:** Yuto Fukuyama, Kimiho Omae, Yasuko Yoneda, Takashi Yoshida, Yoshihiko Sako

**Affiliations:** Laboratory of Marine Microbiology, Graduate School of Agriculture, Kyoto University, Kitashirakawa Oiwake-cho, Sakyo-ku, Kyoto, Japan

## Abstract

*Carboxydothermus* spp. are some of the most studied carbon monoxide–oxidizing anaerobic thermophiles. For further investigation into the carbon monoxide metabolism of *Carboxydothermus* spp., we report here the draft genome sequences of the hydrogenogenic carboxydotrophs *Carboxydothermus pertinax* (2.47 Mb; G+C content, 40.7%) and *C. islandicus* (2.39 Mb; G+C content, 42.0%).

## GENOME ANNOUNCEMENT

*Carboxydothermus* spp. are carbon monoxide (CO)–oxidizing, anaerobic, Gram-positive thermophiles from the family *Thermoanaerobacteriales* (1, 2). To date, five *Carboxydothermus* spp. (*C. hydrogenoformans*, *C. ferrireducens*, *C. siderophilus*, *C. islandicus*, and *C. pertinax*) have been described. Except for *C. ferrireducens*, these bacteria produce hydrogen for their growth via CO oxidation (hydrogenogenic carboxydotrophy) (2–6). *C. hydrogenoformans* has been studied as a model of hydrogenogenic carboxydotrophy and its genome contains five genes of CO dehydrogenase (CODH), a key enzyme of CO metabolism (7, 8). For further investigation into the CO metabolism of *Carboxydothermus* spp., we report here the draft genome sequences of *C. pertinax* and *C. islandicus* (2, 6).

Genomic DNA of *C. pertinax* and *C. islandicus*, extracted using previously described NaOH methods (9), were subjected to sequencing with the Illumina MiSeq platform (Illumina Inc., San Diego, CA, USA) using the 2 × 150-bp paired-end approach, generating 3,839,951 and 6,663,869 paired-end reads, respectively. High-quality reads (Phred score above Q20 for 80% of the bases) were assembled into contigs using Velvet version 1.2.07 or version 1.2.10 software (10). The assembled contigs were subjected to the Microbial Genome Annotation Pipeline (MiGAP; http://www.migap.org/index.php/en) (11) to predict open reading frames (ORFs), followed by manual curation. Subsequently, the protein sequences were annotated using BLASTp searches (12, 13) against nonredundant protein sequences in the NCBI database.

The draft genomes of *C. pertinax* and *C. islandicus* were assembled into 96 (2.47 Mb) and 142 (2.39 Mb) contigs, respectively. These draft genomes have an average G+C content of 40.7% and 42.0%, respectively. The numbers of predicted ORFs were 2,577 and 2,480, respectively.

We identified four CODH gene clusters (corresponding to CODH-II to CODH-V of *C. hydrogenoformans*) (8) in *C. pertinax* and five CODH gene clusters (corresponding to CODH-I to CODH-V of *C. hydrogenoformans*) (8) in *C. islandicus*. The functions of the five CODHs in *C. hydrogenoformans* have been predicted according to empirical evidence and/or the genomic contexts of their genes (*cooS*) (8, 14, 15): CODH-I for energy conversion conjugated with energy-converting hydrogenase (ECH); CODH-II for NAD (P) H generation; CODH-III for carbon fixation in the Wood–Ljungdahl pathway; CODH-IV for oxidative stress response; and CODH-V for unknown function. To our knowledge, CODH gene clusters for energy conversion are adjacent to or located near the ECH gene cluster on the genomes of hydrogenogenic carboxydotrophs, and complexes of these gene products are considered to be responsible for hydrogenogenic CO metabolism (16, 17). The CODH/ECH gene cluster was conserved in *C. islandicus*, as in other hydrogenogenic carboxydotrophs, whereas the genome of *C. pertinax* contained an ECH gene cluster but not the CODH/ECH gene cluster. In addition, *cooS*, which encodes CODH-I, was not amplified when using a specific PCR primer set (forward primer, 5′GCGGCGCGGGATTCCTTTAG3′; reverse primer, 5′AAGCCCGGCTGCCTTTCCTA3′). These results suggested that for *C. pertinax* complexes of gene products from the ECH gene cluster and its physically unlinked *cooS* were responsible for hydrogenogenic CO metabolism.

### Accession number(s).

The draft genome sequences of *C. pertinax* and *C. islandicus* have been deposited in the DNA Data Bank of Japan under the GenBank accession numbers BDJK01000000 and BDJL01000000, respectively.
